# Japanese Legacy Cohort Studies: The Hisayama Study

**DOI:** 10.2188/jea.JE20180150

**Published:** 2018-11-05

**Authors:** Toshiharu Ninomiya

**Affiliations:** Department of Epidemiology and Public Health, Graduate School of Medical Sciences, Kyushu University, Fukuoka, Japan

**Keywords:** epidemiology, cohort study, cardiovascular disease, dementia, diabetes

## Abstract

The Hisayama Study is a population-based prospective cohort study designed to evaluate the risk factors for lifestyle-related diseases, such as stroke, coronary heart disease, hypertension, diabetes, and dementia, in a general Japanese population. The prospective follow-up surveys have been conducted in subjects aged 40 or older since 1961. Notable characteristics of this study include its high participation rate (70–80% of all residents aged 40 or older), high follow-up rate (99% or over), and high autopsy rate (approximately 75% of deceased cases). The Hisayama Study has provided valuable evidence of secular change in the prevalence and incidence of several lifestyle-related disease and their risk factors. The study has thereby contributed to elucidation of the preventive strategies for lifestyle-related disease. Research efforts in this cohort are ongoing and will provide additional data for the improvement of human health and longevity.

## ORIGIN OF THE COHORT

**Figure 1.**
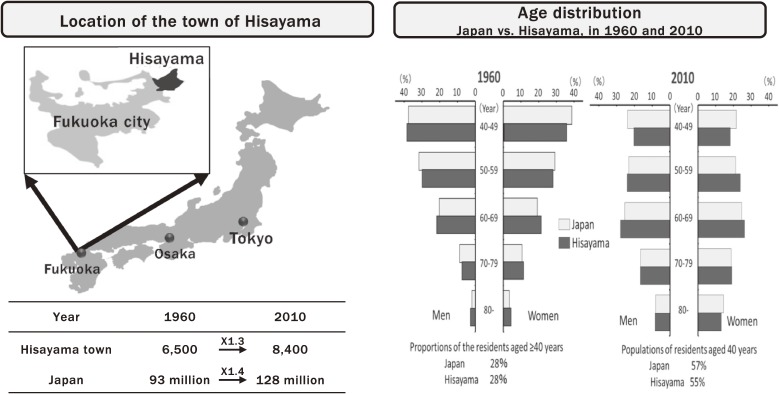
The town of Hisayama

The Hisayama Study is a population-based prospective cohort study that has been conducted in the town of Hisayama, Japan since 1961 and is still ongoing. The town of Hisayama is a typical rural area in Kasuya-gun, Fukuoka Prefecture, adjacent to the north-east of Fukuoka City, Kyushu Island, Japan. Its land area is 37.7 square kilometers and consists of several hills and farm lands (Figure 1). The reasons why the town of Hisayama were selected for this study were as follows: 1) the population of the town of Hisayama was approximately 6,800 in the 1960s (it is 8,500 at present), and the residents of Hisayama have almost the same age and occupational distribution as the national average, making it a representative sample for the typical Japanese population; 2) the town of Hisayama is located within 10 miles from Kyushu University, allowing us to keep close communications with residents; 3) annual variations of the town’s population have been small, enabling a follow-up survey with a low rate of loss to follow-up; and 4) the town mayor and general practitioners in this town are strongly supportive of the epidemiological research to improve the health condition of residents.^1^

The Hisayama Study was initiated in order to clarify certain doubts regarding national mortality statistics for the Japanese population. In the 1960s, stroke was the leading cause of death in Japan. The mortality rate from brain hemorrhage was especially high, 12.4-fold greater than that from brain infarction, which was exceedingly high among 33 countries. In 1961, Goldberg and Kurland pointed out that the apparent difference in the mortality rate from brain hemorrhage in Japan was due to environmental effects or artifacts, including diagnosis, rather than to ethnic characteristics.^2^ They discussed the necessity of a more accurate diagnostic approach to determine the stroke subtypes.

The Hisayama Study was initiated by Professor Shibanosuke Katsuki, who led the 2^nd^ Department of Internal Medicine, Kyushu University (now the Department of Medicine and Clinical Science, Graduate School of Medical Sciences), in order to elucidate the accurate incidence and mortality of stroke and its subtypes in a community-dwelling Japanese population. Most of the epidemiological studies in Japan and other countries are performed on the basis of death certificates and clinical records. However, at the time of the study launch, the reliability of the diagnosis of stroke subtypes based on physical examinations and death certificates in classifying disease was not very certain in the absence of postmortem examinations, because imaging diagnosis methods—namely, computed tomography (CT) and magnetic resonance imaging (MRI) scans—had not yet been developed. Thus, the design of the study called for autopsy examinations in all deceased cases in Hisayama in order to accurately diagnose stroke and its subtypes. The results based on autopsy data demonstrated that the mortality from brain hemorrhage was only about 1.1-times higher than that of brain infarction, and misdiagnosis on death certificates was not as uncommon as previously thought.

The Hisayama Study was continued under four professors of the 2^nd^ Department of Internal Medicine, Kyushu University: Professor Teruo Omae in 1971–1984, Professor Masahiro Fujishima in 1984–2000, Professor Mitsuo Iida in 2001–2010, and Professor Takanari Kitazono in 2011–present. The study was managed during this period by Dr. Yasuo Hirota, Dr. Yukimori Takeshita, Dr. Kazuo Ueda, Dr. Yutaka Kiyohara, and Toshiharu Ninomiya. The major research efforts of this study were transferred to the Department of Environmental Medicine in 2006–2016 and the Department of Epidemiology and Public Health in 2016–present, when Dr. Kiyohara and Ninomiya were appointed as professors of the respective departments, but the research procedures remained unchanged. This study was also conducted with the support of the pathological and clinical departments at Kyushu University and of general practitioners and the staff of the Division of Health and Welfare in the town of Hisayama.

## SCOPE AND FEATURES

**Figure 2.**
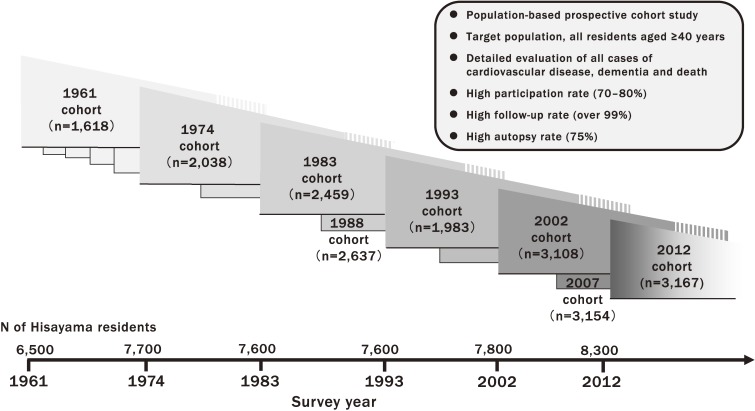
The characteristics of the Hisayama Study

The Hisayama Study is a population-based prospective cohort study designed to evaluate the risk factors for lifestyle-related diseases, such as stroke, coronary heart disease, hypertension, diabetes, and dementia, in a general Japanese population. The prospective follow-up surveys have been conducted in subjects aged 40 years or older since 1961, because the frequency of lifestyle-related disease is expected to increase stepwise or abruptly after the age of 40. To date, cohorts have been established in the years 1961, 1974, 1983, 1988, 1993, 2002, 2007, and 2012 (Figure 2). A maximum effort has been made to include 70–80% of all residents aged 40 years or older in each of these cohorts, and each cohort has had a high follow-up rate of 99% or over and used the same follow-up procedures. These prospective follow-up surveys have revealed changes in the daily lifestyles of Japanese, as well as secular trends in the burden of lifestyle-related diseases and their risk factors over the long-term. Moreover, one of the most important characteristics of the study is that the average rate of autopsy examination among decedents in this town has been approximately 75% over the last 57 years. It is well known that autopsy is one of the best ways to accurately determine the cause of death and the disease subtype (eg, dementia). Therefore, to our knowledge, this study would be one of the studies with the highest precision of cohort data in the world. Another key characteristic is that this study is carried out mainly by clinical physicians. This unique approach enables research from the perspectives of both clinical practice and public health.

The major research subjects of this study are cardiovascular diseases (ie, stroke and coronary heart disease), dementia, hypertension, diabetes, metabolic disorders, kidney disease, and gastrointestinal cancer. In addition, the research into the genomic epidemiology of lifestyle-related diseases, including single nucleotide polymorphisms (SNPs), has revealed candidate genes for brain infarction, ulcerative colitis, dementia, and age-related macular degeneration.^3^^–^^6^ In more recent years, the research topics have been diversified to include ophthalmology, dentistry, psychiatry, respiratory disease, and orthopedics.

## SURVEY METHODS

The study participants have been followed-up prospectively via regular health examinations. For participants who do not undergo regular examination or who have moved away from town, mail and telephone follow-ups have been used for approximately the last 50 years.^7^^,^^8^ The health information of participants is also collected through a daily monitoring system established by the study team, local physicians, and the members of the town’s Health and Welfare Office. In this system, the physicians in the study team visit clinics, hospitals, and the town’s office regularly in order to collect information on events of cardiovascular disease and dementia, including suspected cases.

Regular health examinations are repeated every 1–2 years to obtain information on individual health conditions, including cardiovascular events and dementia. Physical and biomedical characteristics—namely, blood pressure, diabetic status, anthropometric measures, electrocardiogram, urinalysis, and blood tests—are collected in the health examinations. Each participant is asked by trained interviewers to complete a self-administered questionnaire covering smoking habits, alcohol intake, and physical activity, educational status, medical history of cardiovascular disease (coronary heart disease, including myocardial infarction and coronary intervention) and other chronic diseases, and any treatments including antihypertensive agents, insulin and oral hypoglycemic agents, and lipid-modifying agents. Blood pressure is measured three times in a recumbent position and/or sitting position using a standard mercury sphygmomanometer (until 2002) or an automated sphygmomanometer (since 2002). Diabetes was diagnosed using an oral glucose tolerance test in subjects with glycosuria in 1961, using fasting and postprandial glucose concentrations in 1974 and 1993, and using 75-g oral glucose tolerance test (OGTT) since 1988, in addition to medical history of diabetes. Body height and weight are measured in light clothing without shoes. Waist circumference is measured at the umbilical level in a standing position by a trained staff member.

With regard to dementia, a two-stage prevalence survey of dementia has been repeated every 5–7 years among the town residents aged 65 years or older since 1985 in the same manner (Table 1).^9^^,^^10^ More than 92% of town residents aged 65 years or older participated in these surveys. In the first screening survey, several neuropsychological tests are performed: the Hasegawa’s Dementia Scale (HDS), the HDS revised version (HDS-R), and the Mini-Mental State Examination (MMSE). Participants are also asked to complete questionnaires regarding activities of daily living, sociodemographic status, and medical conditions. A second screening survey of dementia, including physical and neurological examinations, interviews of the families and attending physicians, and review of the medical records, is carried out by stroke physicians and psychiatrists when a subject is suspected of having new neurological symptoms, including cognitive impairment.

**Table 1.** Demographic characteristics of participants and diagnostic procedures of dementia in five prevalence surveys

	Year of survey				

	1985 (*n* = 887)	1992 (*n* = 1,189)	1998 (*n* = 1,437)	2005 (*n* = 1,566)	2012 (*n* = 1,904)
Age, years, mean (SD)	74 (6)	74 (7)	75 (7)	76 (7)	76 (8)
Women, %	60.2	60.1	60.3	60.9	59.0
Participation rate, %	94.6	96.6	99.7	91.5	93.5
Neuropsychological test	HDS	HDS	HDS-R	HDS-R	HDS-R
		HDS-R		MMSE	MMSE
		MMSE			
Diagnosis of dementia	DSM-III	DSM-III-R	DSM-III-R	DSM-III-R	DSM-III-R

DSM-III, Diagnostic and Statistical Manual of Mental Disorders, third edition; DSM-III-R, Diagnostic and Statistical Manual of Mental Disorders, revised third edition; HDS, Hasegawa’s Dementia Rating Scale; HDS-R, Hasegawa’s Dementia Rating Scale, revised version; MMSE, Mini-Mental State Examination; SD, standard deviation.

The available information about potential cardiovascular events, dementia, and deaths for the study participants is collected and reviewed by physician members of the study. When a participant dies, autopsy is performed at the Department of Pathology of Kyushu University, if consent for autopsy is obtained. All of the event data for cardiovascular disease, dementia, and cause of death are adjudicated on the basis of physical examinations and a review of available clinical information, including medical records, imaging, and autopsy data under the standardized diagnostic criteria throughout the study period.

## DEFINITION OF MAIN OUTCOMES

Cardiovascular disease is defined as development of stroke or coronary heart disease. The definition of stroke is a sudden onset of focal and nonconvulsive neurological deficit lasting more than 24 hours, and the subtype of stroke is classified as either brain infarction, brain hemorrhage, subarachnoid hemorrhage, or undetermined type.^8^ The diagnosis of stroke and its subtypes is diagnosed using medical history, neurological examination, and available clinical data, including brain imaging and autopsy findings. Brain infarction is subclassified according to the Classification of Cerebrovascular Disease III proposed by the National Institute of Neurological Disorders and Stroke,^11^ as well as on the basis of the diagnostic criteria of the Trial of Org 10172 in Acute Stroke Treatment (TOAST) Study^12^ and Cerebral Embolism Task Force.^12^ Coronary heart disease is defined as acute myocardial infarction, silent myocardial infarction, sudden cardiac death within 1 hour after the onset of acute illness, or coronary artery disease followed by coronary artery bypass surgery or angioplasty.^8^ The diagnosis of acute myocardial infarction is based on at least two of the following criteria: 1) typical symptoms, including severe chest pain, dyspnea, or epigastralgia; 2) elevation of cardiac enzymes, more than twice the upper limit of the normal range; 3) electrocardiogram changes, including ST elevation, ST depression, and pathological Q wave; and 4) morphological changes, including asynergy of left ventricular wall on echocardiography and/or coronary angiography, persistent regional myocardial perfusion defect on cardiac scintigraphy, or myocardial necrosis or scars more than 1 cm in diameter at autopsy. Silent myocardial infarction is defined as myocardial scarring without any historical indication of clinical symptoms or abnormal cardiac enzyme changes.

Dementia is diagnosed based on clinical symptom according to the guidelines of the Diagnostic and Statistical Manual of Mental Disorders, third edition (DSM-III) in 1985^13^ and those of the DSM-III revised version (DSM-III-R) since 1992.^14^ The subtype of dementia is classified as Alzheimer’s disease (AD) or vascular dementia (VaD) according to the National Institute of Neurological and Communicative Disorders and Stroke and the Alzheimer’s Disease and Related Disorders Association criteria,^15^ and the National Institute of Neurological Disorders and Stroke-Association Internationale pour la Recherche et l’Enseignement en Neurosciences criteria,^16^ respectively.

## MAJOR PUBLICATIONS

### Cardiovascular disease

**Figure 3.**
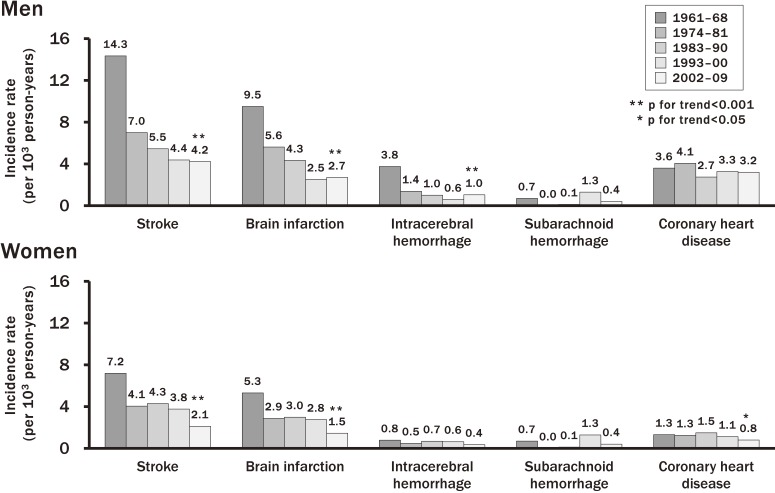
Secular trends in the incidence of stroke and coronary heart disease in the town of Hisayama. Data from 5 Hisayama cohorts of patients aged ≥40 years, with 7-year follow-up and age-adjustment (cited from reference 11)

**Figure 4.**
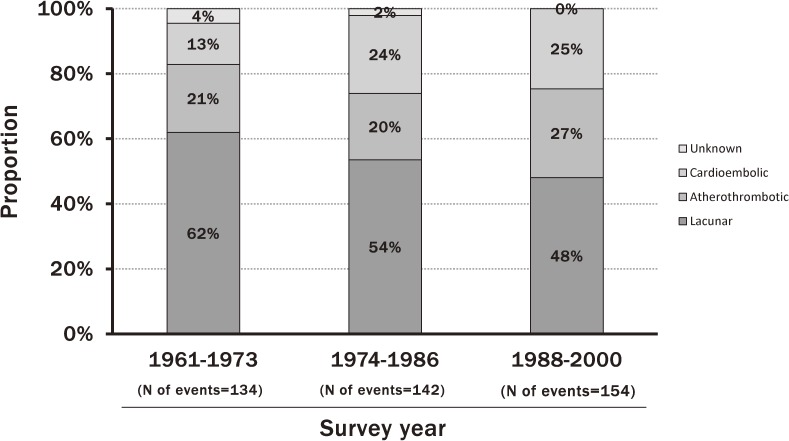
Trends in the proportion of brain infarction subtypes in the town of Hisayama. Data from 3 Hisayama cohorts of patients aged ≥40 years, with 13-year follow-up (modified by reference 19)

Cardiovascular disease is one of the leading causes of death worldwide. According to the vital statistics, Japanese populations had a higher mortality from stroke and a lower mortality from coronary heart disease than Western populations in the 1960s, and the mortality from stroke started to decrease in the 1970s.^17^ Lifestyle changes and improvement of prevention and management of cardiovascular risk factors in Japanese are likely to affect the incidence and mortality of cardiovascular disease. However, there have been few studies estimating the secular changes in the incidence of cardiovascular disease and the survival rate after onset of cardiovascular disease in Japan over the past half century. The Hisayama Study investigated this issue using the 7-year follow-up data from five cohorts, which were established in each decade from the 1960s to the 2000s and consisted of residents aged 40 years or older in a Japanese community (Figure 3). The age-adjusted incidence of stroke decreased steeply, by 51% in men and 43% in women, from the 1960s to the 1970s, but this decreasing trend slowed down in the subsequent decades.^8^ Similar downward trends in the incidence of brain infarction in both sexes and the incidence of intracerebral hemorrhage in men were observed. In this study, the secular changes in the proportion of subtypes of brain infarction were also investigated using 13-year follow-up data from 3 cohorts in 1961, 1974, and 1988 (Figure 4). The age-adjusted incidence of lacunar infarction declined significantly, whereas the incidences of atherothrombotic and cardioembolic infarction did not change during this period. As a consequence, the proportion of lacunar infarction decreased and the proportion of atherothrombotic and cardioembolic infarction increased.^18^ With regard to coronary heart disease, there was no significant secular change in the age-adjusted incidence of coronary heart disease over the period in men, but it decreased significantly from the 1980s to the 2000s in women.^11^ However, the incidence of acute myocardial infarction did not decrease in either sex. The 5-year survival rate of stroke after onset improved significantly from the 1960s to the 1980s and improved slightly thereafter. The survival rate of acute myocardial infarction in the 2000s significantly improved compared with that in the 1960s.

This study also provided a number of epidemiological findings concerning the influence of several risk factors on the development of stroke and coronary heart disease—namely, hypertension, diabetes, hyperlipidemia, metabolic syndrome, chronic kidney disease, and systemic inflammation.^19^^–^^24^ From the 1960s to the 2000s, the management of blood pressure improved significantly and the frequency of current smoking decreased in our study population, whereas the prevalence of metabolic disorders, such as glucose intolerance, hypercholesterolemia, and obesity, increased significantly with time.^8^ These changes in the status of cardiovascular risk factors would have strongly affected the risk of cardiovascular disease and its subtypes during the past half century among Japanese.

### Diabetes mellitus

The rising prevalence of diabetes mellitus is a great public health concern, because diabetes mellitus can lead to complications in several organ systems, including both macro- and micro-vascular complications. The number of patients with diabetes mellitus has been rapidly growing worldwide, especially in Asia, probably owing to the aging of the population, economic development, and an increasingly Westernized lifestyle.^25^ Reliable estimations of secular changes in the prevalence of diabetes mellitus are necessary to develop effective strategies for prevention and management of diabetes mellitus. However, there are limited data addressing the trends in the prevalence of diabetes mellitus, which is defined using a 75-g OGTT, in general Asian populations.

**Figure 5.**
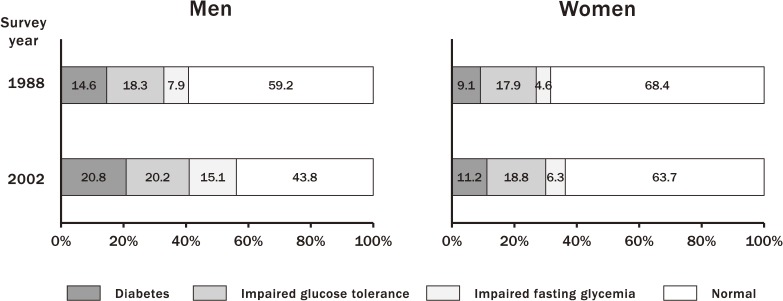
Secular trends in the prevalence of glucose intolerance diagnosed by oral glucose tolerance test. Data are shown for Hisayama residents in 1988 (*n* = 2,490) and 2002 (*n* = 2,852), aged 40–79 years, with age-adjustment (cited from reference 27)

The Hisayama Study investigated secular trends in the prevalence of diabetes mellitus and prediabetes, defined using the OGTT, from 1988 to 2002 in a general Japanese population.^26^ The results showed that the secular trends in the age-adjusted prevalence of diabetes mellitus and impaired fasting glycemia (IFG) increased over a 14-year period in both sexes (diabetes: 14.6% in 1988 to 20.8% in 2002, *P* < 0.001 for men; 9.1% in 1988 to 11.2% in 2002, *P* = 0.002 for women; impaired glucose tolerance [IGT]: 7.9% in 1988 to 15.1% in 2002, *P* < 0.001 for men; 4.6% in 1988 to 6.3% in 2002, *P* = 0.049 for women), whereas the increasing trend in the prevalence of IGT did not reach a statistically significant level in either sex (18.3% in 1988 to 20.2% in 2002, *P* = 0.30 for men; 17.9% in 1988 to 18.8% in 2002, *P* = 0.26 for women) (Figure 5). The age-specific prevalence of diabetes mellitus increased dramatically and significantly over time in men aged 60–79 years and in women aged 70–79 years. These rising trends in the prevalence of diabetes mellitus were likely to be attributable to the increasing trends in the frequency of obesity and the decline in physical activity, especially in elderly subjects.

This study identified several risk factors for the development of diabetes mellitus and established risk score models for predicting future incidence of diabetes. This risk scoring model included age, sex, family history of diabetes, abdominal circumference, body mass index, hypertension, regular exercise, and current smoking, in addition to fasting plasma glucose levels.^27^ The predictive ability of this risk scoring model was good, with c-statistics of 0.772. This model would help to identify people with higher risk of diabetes mellitus, and could thereby reduce the burden of diabetes among Japanese by encouraging high-risk individuals to improve their lifestyles.

### Dementia

**Figure 6.**
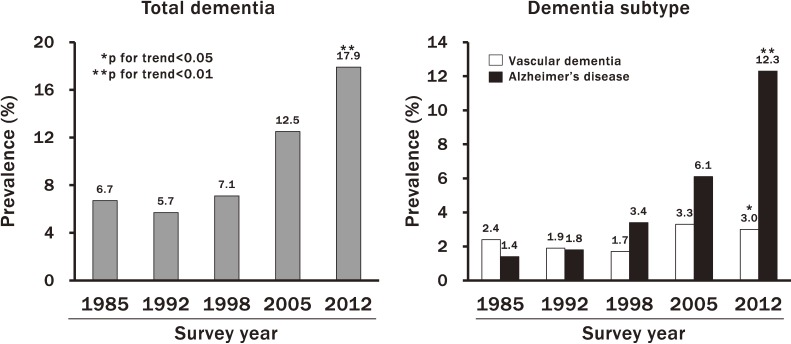
Trend in the prevalence of total dementia and its subtypes in the town of Hisayama. Data are shown for Hisayama residents aged ≥65 years, without adjustment (cited from reference 33)

The Hisayama Study revealed that the prevalence of dementia, especially AD, increased greatly over the past quarter century in a general population of elderly Japanese. The unadjusted prevalence of total dementia increased significantly with time (6.7%, 5.7%, 7.1%, 12.5%, and 17.9% for the years 1985, 1992, 1998, 2005 and 2012, respectively; *P* for trend <0.01) (Figure 6).^10^ With regard to dementia subtypes, an increasing trend in the prevalence of AD was observed (1.4%, 1.8%, 3.4%, 6.1%, and 12.3%, respectively; *P* for trend <0.01), while the prevalence of VaD showed a decreasing trend between 1985 and 1998 and thereafter an increasing trend (2.4%, 1.9%, 1.7%, 3.3%, 3.0%, respectively; *P* for trend = 0.02). Aging of the population is a major cause of the increase in the prevalence of dementia. However, after controlling for the confounding effects of aging, the upward trends in the prevalence of total dementia and AD remained significant, while the increasing trend in the prevalence of VaD disappeared. These findings suggest that the burden of dementia, especially AD, has increased rapidly over the past 2 decades in Japan. The increasing trend in the incidence of total dementia in Japan may be related to the recent increase in the burden of metabolic disorders, such as diabetes, or the spread of westernized lifestyle behaviors, such as lack of exercise, which have been associated with higher risk of AD.^28^^,^^29^

**Figure 7.**
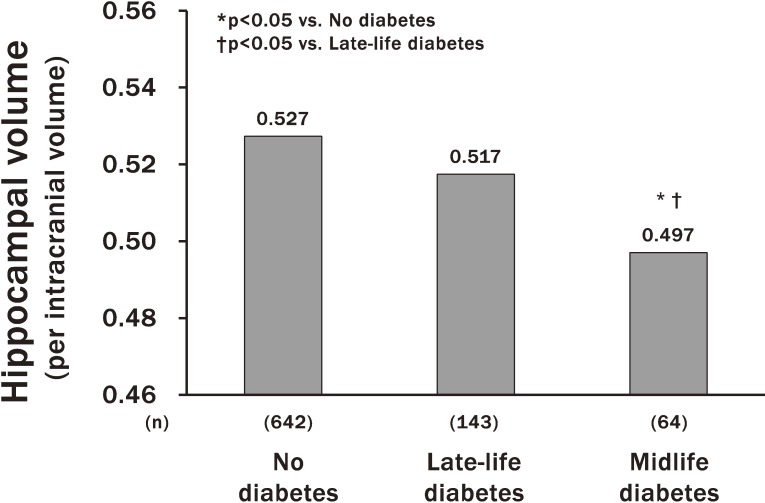
Association between diabetes mellitus and hippocampal atrophy. The values were adjusted for age, sex, education level, hypertension, serum total cholesterol, body mass index, smoking habits, alcohol intakes, regular exercise and cerebrovascular disease (cited from reference 37)

The Hisayama Study has clarified several risk factors and protective factors for dementia—namely, hypertension, diabetes mellitus, smoking habits, dietary pattern, and lack of regular exercise.^30^^–^^34^ In particular, a report on diabetes mellitus as a risk factor for AD has attracted considerable attention.^31^ In this report, diabetes mellitus was associated with a 1.74-fold (95% confidence interval [CI], 1.19–2.53) greater risk of total dementia. With regard to subtypes of dementia, subjects with diabetes mellitus had a 2.05-fold (95% CI, 1.18–3.57) higher risk of AD than those with normal glucose tolerance. Moreover, this study showed that longer duration of diabetes was significantly associated with lower hippocampal volume.^35^ Thus, subjects with diabetes in midlife had a greater risk of hippocampal atrophy than those with diabetes in late life (Figure 7). These findings provide confirmatory evidence that diabetes mellitus is a significant risk factor for AD. Although the mechanisms underlying these findings are not yet fully clarified, the optimal management of diabetes mellitus as early as possible in the life cycle may be important to prevent late-life dementia.

It is hoped that new and effective drugs for dementia will be developed, but no definitive evidence of any effective pharmacological treatment for dementia exists so far. The prevention and optimal management of risk factors, such as hypertension and diabetes, tobacco cessation, regular exercise, and favorable diets are needed as early as possible in the life cycle—well before any cognitive impairment becomes manifest—for the effective prevention of late-life dementia. Further research should attempt to explore effective preventive and/or therapeutic strategies against dementia.

### Functional disability

The Hisayama Study investigated the prevalence of functional disability and its causes among 1,566 residents aged 65 years or older in 2005.^36^ Activity of daily living (ADL) status was defines as a Barthel Index score of ≤95 and was classified categorized into the three following levels: slight dependence (a Barthel Index score of 95), moderate/severe dependence (a score of 25–90), and total dependence (a score of 0–20, which corresponds approximately to a bedridden state). The causes of functional disability were determined using all available past clinical information, including medical records and findings from neurologic examination and brain imaging, which were collected using the follow-up system of the Hisayama Study. As a result, the prevalence of disability was 20% among the participants, which increased with age, with a doubling in prevalence for every 5-year increment. The proportions of subjects with slight, moderate/severe, and total dependence were 25.4%, 49.8%, and 24.8%, respectively. With regard to the causes of functional disability, dementia accounted for 32.5%, stroke for 13.5%, orthopedic disease for 26.0%, and other disease for 28.0% of cases. Approximately 60% of subjects with total dependence were caused by dementia in both sexes. In subjects with slight or moderate/severe dependence, stroke was the most common cause of disability in men and orthopedic disease in women. These findings suggest that functional disability is common and dementia is a major cause of disability among a community-dwelling elderly population in Japan.

## HISTORICAL IMPACT ON GLOBAL OR LOCAL HEALTH

The Hisayama Study has provided valuable insights into the prevalence, incidence, prognosis, and risk factors for a broad range of lifestyle-related diseases, including cardiovascular disease, diabetes, and dementia, over half a century. Moreover, the findings from this study have been taken up in many guidelines in Japan, such as Japanese guidelines for stroke,^37^ coronary heart disease,^38^ hypertension,^39^ diabetes,^40^ atherosclerosis,^41^ and dementia^42^ and are cited in several scientific reviews and meta-analyses. This study has contributed to a reduction in the risk of cardiovascular diseases, especially stroke, in Japan through the dissemination of information regarding the importance of blood pressure control and tobacco cessation. However, the recent rise in the burdens of diabetes and dementia is now an urgent matter of public health. The results from the Hisayama Study will help to elucidate the preventive strategies for both these conditions as well. We will continue our research efforts to provide best research evidence to improve human health and longevity.
